# Controlled Photocatalytic Reduction of CO_2_ by Precise Atomic‐Level Interface Modification and Engineering of Silver Nanoclusters

**DOI:** 10.1002/advs.202516096

**Published:** 2025-10-24

**Authors:** Hangmin Xu, Xiang Liu, Ganghua Zhou, Chuanzhou Bi, Qing Liu, Weiyi Jiang, Bin Wang, Xingwang Zhu, Paul K. Chu, Xiaozhi Wang

**Affiliations:** ^1^ School of Mechanical Engineering, College of Environmental Science and Engineering Yangzhou University Yangzhou 225009 P. R. China; ^2^ Department of Physics, Department of Materials Science and Engineering, and Department of Biomedical Engineering City University of Hong Kong Kowloon Hong Kong 999077 P. R. China

**Keywords:** Ag nanoclusters, atomic‐level precision, CO_2_ activation, electron potential well, nanoclusters

## Abstract

The emission of carbon dioxide (CO_2_) and other greenhouse gases has raised serious environmental concerns, and artificial photosynthesis is a promising approach to reducing the carbon footprint. The primary challenge for photocatalytic systems is how to optimally separate interfacial charges, while the hydrogen evolution reaction limits the selectivity of products in the photocatalytic reduction of CO_2_. Herein, highly stable Ag_44_ nanoclusters (Ag_44_ NCs) protected by thiol salt ligands are prepared with atomic‐level precision. The ultra‐small Ag_44_ NCs shorten the distance for electrons to migrate from the bulk phase to the surface and accelerate interfacial charge transfer. Furthermore, the molecule‐like properties of Ag_44_ NCs broaden the light absorption range of the semiconducting substrate, and quantum confinement rendered by Ag_44_ NCs produces a potential well, which promotes electron aggregation and generates a long‐range ordered electric field to transfer electrons directionally. Since the electrostatic repulsion of positively charged Ag_44_ NCs hinders electron transfer and proton coupling, the hydrogen evolution reaction is inhibited.

## Graphical abstract

Ag_44_ nanoclusters are anchored on the CdS surface via low‐temperature electrostatic deposition. This facilitated the directed migration of electrons at the interface, thereby effectively suppressing the hydrogen evolution reaction and promoting the highly selective reduction of CO_2_ to carbon monoxide (CO).

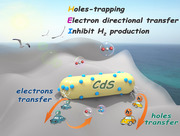

## 1 Introduction

Semiconductor‐catalyzed artificial photosynthesis is an effective and sustainable strategy to abate the greenhouse effect. Nevertheless, the inefficient utilization of electrons and the limited CO_2_ activation capacity of semiconductors have posed significant challenges to industrial implementation.^[^ ^1^ ^]^ Therefore, how to achieve directional migration of electrons on semiconductors as well as effective adsorption and activation of CO_2_ is the key for artificial photosynthesis.^[^ ^2^, ^3^ ^]^ In order to facilitate the directional transfer of electrons in semiconductors, it is essential to optimize their physical and chemical properties. Precious metal‐loaded semiconductors are capable of overcoming the physicochemical properties inherent to semiconductors to produce surface active sites for better directional electron transfer.^[^ ^4^, ^5^ ^]^ In particular, metal nanoclusters have garnered considerable interest due to their unique catalytic properties arising from quantum confinement,^[^ ^6^, ^7^ ^]^ surface geometry,^[^ ^8^, ^9^ ^]^ and molecule‐like structure.^[^ ^10^, ^11^ ^]^ The quantum confinement effect leads to the formation of discrete electronic energy levels in Ag_44_ NCs, resulting in electron confinement bound by nanoscale potential wells. Electrons of the metal nanoclusters with molecule‐like properties can readily interact with adsorbates to facilitate CO_2_ adsorption and activation.^[^ ^12^ ^]^ However, the principal disadvantage of metal nanoclusters is their structural instability, as they are susceptible to agglomeration at high temperatures and in air. To address this challenge, two key strategies have been proposed. First, suitable ligands can be selected to safeguard the metal core from external influences.^[^ ^13^ ^]^ Second, the metal nanoclusters are uniformly anchored to the crystalline materials by exploiting the confinement effect.^[^ ^14^ ^]^ The rod‐like CdS typically exhibits high crystallinity, thus offering the potential to anchor metal nanoclusters on the surface and to prevent aggregation of metal nanoclusters. In fact, the drawbacks of CdS photo‐corrosion have been mitigated when CdS contains metal nanoclusters protected by thiolate ligands.^[^ ^9^, ^15^ ^]^

In the process of CO_2_ photoreduction, many reactions occur concurrently. Among them, the hydrogen evolution reaction (HER) consumes photogenerated electrons and decreases the efficiency of CO_2_ reduction. In the oxygen evolution reaction, AgNO_3_ is typically selected as a sacrificial agent to circumvent the competition with HER.^[^ ^16^ ^]^ It is therefore of great significance to prepare atomically precise Ag nanoclusters and investigate the electron transfer mechanism in the photocatalytic reduction of CO_2_ during the interactions with CdS nanorods.

Herein, in light of the considerable benefits associated with Ag_44_ NCs, in situ anchoring is adopted to develop the Ag_44_ NCs/CdS artificial photocatalytic system to introduce Ag_44_ NCs to CdS. The low‐temperature process produces a graded and seamless interface in the catalysts. When photogenerated electrons in the semiconductor are excited to the conduction band, they promptly migrate to the Ag_44_ NCs by directional electron transfer. Concurrently, the electron potential well of Ag_44_ NCs facilitates electron aggregation and ensures a consistent supply of electrons for CO_2_ activation. Ultimately, the positively charged silver functions as an electron acceptor to impede the transfer of single electrons and the generation of hydrogen.

## 2 Results and Discussion

Theoretical models of CdS, Ag_44_ NCs/CdS (ACS), and Ag nanoparticles/CdS (Ag NPs/CdS, APS) are first constructed (Figure S1, Supporting Information). Through the process of structural optimization of the theoretical computational model, it was discovered that the energy change in ACS is significantly higher than that in CdS and APS (Table S1, Supporting Information), indicating that the introduction of Ag_44_ NCs results in strong interactions between the internal atoms. After the deposition of Ag_44_ NCs and Ag NPs on CdS, the work functions (Figure S2a‐c, Supporting Information) of CdS, ACS, and APS are calculated to be 5.83, 4.18, and 5.13 eV, respectively, suggesting that after the introduction of Ag, photogenerated electrons on CdS can transfer to the Ag sites.^[^ ^17^, ^18^ ^]^ Our calculations disclose that Ag_44_ NCs are more effective than Ag NPs in gathering electrons, thus making Ag_44_ NCs an electron potential well. By studying the density of states (DOS, Figure S3a‐c, Supporting Information), the incorporation of the metallic Ag enhances the conductivity of CdS, expedites the transfer of photogenerated electrons to the electron potential well, and reduces the band structure of CdS to enhance light absorption of CdS, thereby making it possible for CdS to excite more electrons for the reactions (Figure S4a‐c, Supporting Information). To corroborate this point, the light absorption capacity of the catalyst is calculated. As shown in Figure S5a‐c (Supporting Information), the light absorption spectra of ACS and APS exhibit marked enhancement in comparison with CdS. Furthermore, ACS exhibits a greater light absorption capacity than APS. The primary reason is the small size of Ag_44_ NCs. This enables the continuum density of states to be divided into discrete energy levels, thereby exhibiting molecule‐like optical absorption characteristics in the visible to near‐infrared optical range.^[^ ^19^ ^]^

**Figure 1:**
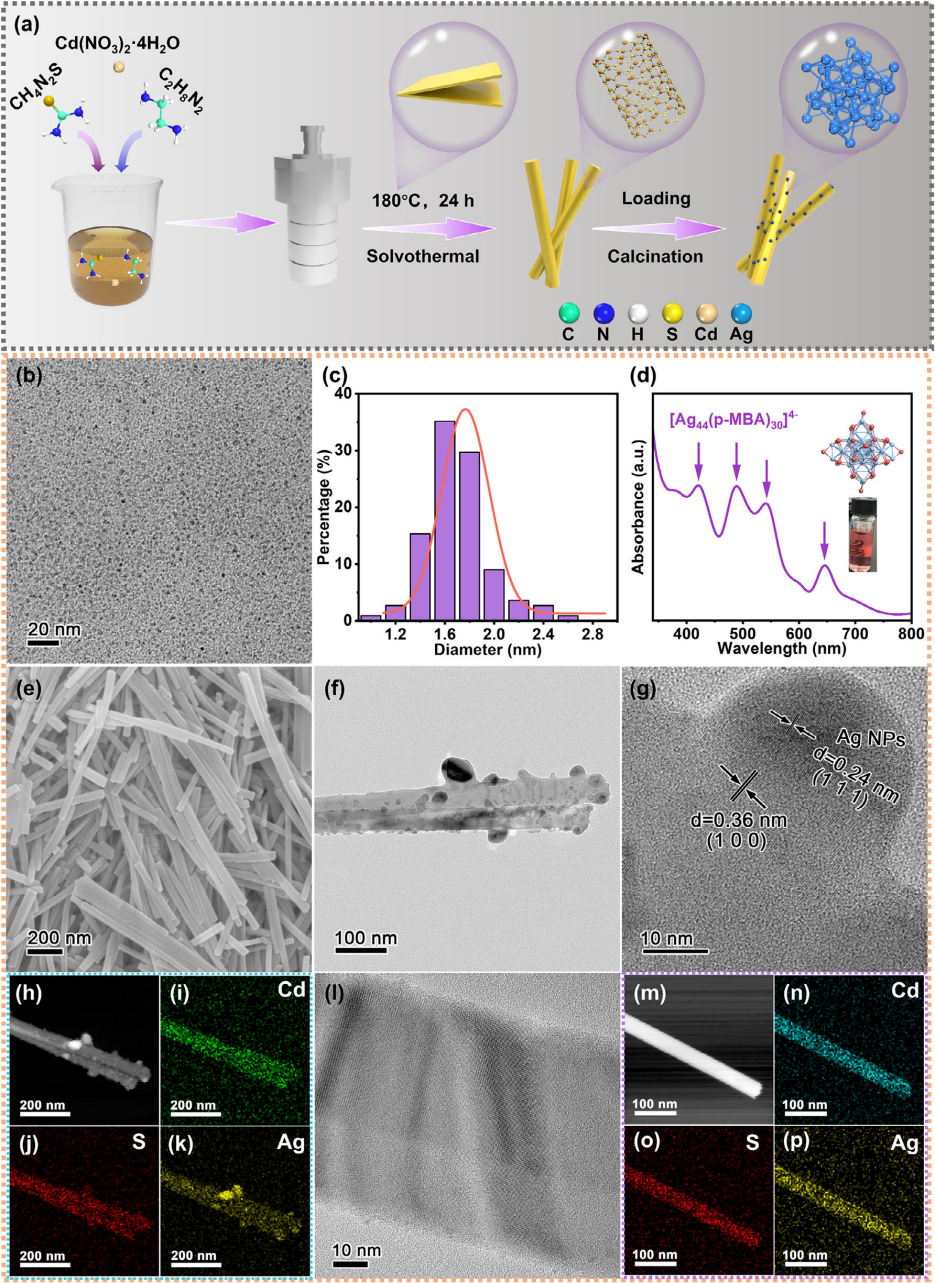
a) Schematic diagram of the ACS catalyst synthesis process; b) HR‐TEM image of Ag_44_ NCs (Inset indicates the particle size distribution of Ag_44_ NCs); c) Particle size distribution of Ag_44_ NCs; d) Liquid UV‐vis spectroscopy spectrum of Ag_44_ NCs; e) SEM image of ACS‐2; f) TEM image; g) HR‐TEM image; h) STEM image and i‐k) EDS elemental maps of APS; l) HR‐TEM image, m) STEM image, and n‐p) EDS elemental maps of ACS‐2.

The CdS nanorods are synthesized by a one‐pot method at 180 °C. The Ag_44_ NCs/CdS (ACS‐X, X = 1, 2, or 3. The “X” value denotes the amount of Ag_44_ NCs solution added to the CdS surface, with 1, 2, and 3 corresponding to 0.05 mL, 0.3 mL, and 0.6 mL, respectively.) samples were prepared by low‐temperature electrostatic deposition of Ag_44_ NCs on CdS nanorods, as illustrated in **Figure** **1**a. The Ag_44_ NCs shown in Figure S6a (Supporting Information) have a uniform distribution in the high‐resolution transmission electron microscopy (HR‐TEM) image (Figure 1b). They have a size of approximately 1.6 nm (Figure 1c). In order to assure the atomic‐level precision of the synthesis, the UV‐vis spectra are acquired from the liquid. Figure 1d exhibits peaks at 421, 489, 541, and 646 nm, and the protonated solution has a mauve color, suggesting that the Ag NCs are composed of 44 Ag atoms.^[^ ^20^, ^21^ ^]^ Moreover, the Ag_44_ NCs can be stored at a low temperature for a long period of time before being protonated. Only a small degree of Ag_44_ NCs agglomeration is observed in Figure S6b (Supporting Information). As shown in Figure S6c (Supporting Information), the intensity of Ag_44_ decreases slightly due to the agglomeration of nanoclusters, consequently demonstrating good stability of Ag_44_ NCs prepared using the thiolate ligand. The SEM (Figure 1e) and TEM (Figure S7a, Supporting Information) images reveal that ACS‐2 has a rod‐like structure. CdS shows high crystallinity in the HR‐TEM image (Figure S7b, Supporting Information) with the (100) crystal plane for effective crystal confinement. As shown in Figure 1f,g, the large‐sized Ag NPs expose the (111) crystal faces and attach strongly to the CdS nanorods. The elemental distribution maps (Figure 1h‐k) demonstrate that the Ag NPs are uniformly distributed on the CdS nanorods. The HR‐TEM image (Figure 1l) of ACS‐2 shows ∼1.6 nm quantum dots without a lattice on CdS, and Figure 1m‐p ascertains the quantum dots are Ag_44_ NCs distributed uniformly on CdS.

**Figure 2:**
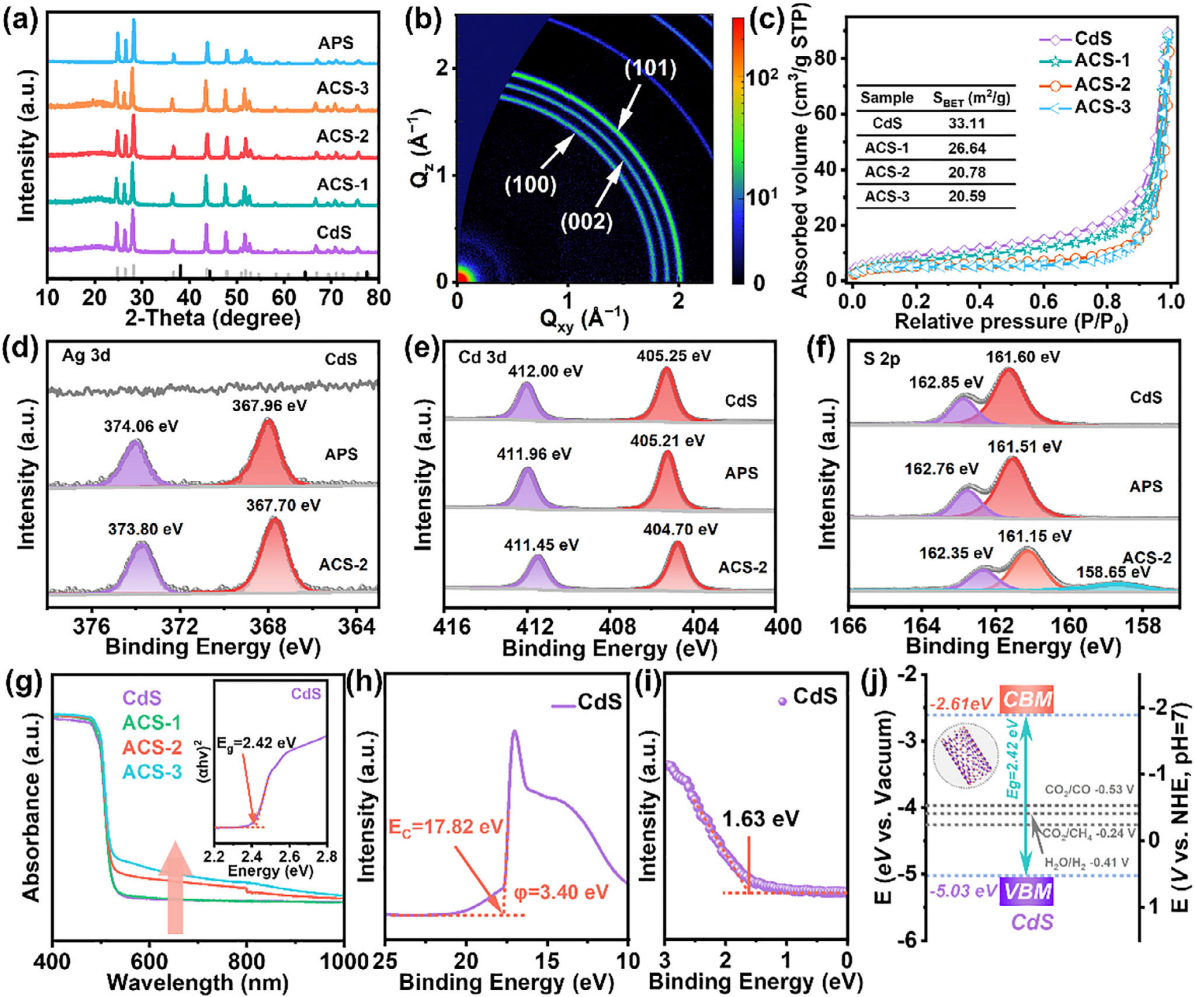
a) XRD spectra; b) 2D GI‐WAXS patterns of ACS‐2; c) N_2_ adsorption and desorption isotherms; High‐resolution spectra of d) Ag 3*d*, e) Cd 3*d*, and f) S 2*p* of CdS, ACS‐2, and APS; g) UV‐vis diffuse reflectance spectra (Inset shows the bandgap of CdS); h) UPS diagram of CdS; i) XPS‐VB plot of CdS; j) Band structure of CdS.

X‐ray diffraction (XRD, 2D GI‐WAXS), N_2_ adsorption and desorption assessment, and X‐ray photoelectron spectroscopy (XPS) are conducted to investigate the structure of the materials. The peaks corresponding to CdS in XRD align with the standard card (No.06–314), but no signal from Ag is detected,^[^ ^22^ ^]^ perhaps due to the small Ag concentration and small size of the Ag_44_ NCs and Ag NPs (**Figure** **2**a).^[^ ^23^ ^]^ Additionally, no Ag signals are observed by 2D GI‐WAXS (Figure 2b).^[^ ^24^ ^]^ As the amount of Ag_44_ NCs increases, the specific surface area decreases because the loading of Ag_44_ NCs on CdS hides some of the original materials (Figure 2c). In the XPS survey spectrum (Figure S8, Supporting Information), Ag is detected from ACS‐2 and APS, confirming the presence of Ag NPs and Ag_44_ NCs on CdS. In the high‐resolution spectrum of Ag 3*d* (Figure 2d), the peaks at 374.06 eV and 367.96 eV of APS and 373.80 eV and 367.70 eV of ACS‐2 correspond to Ag 3*d*_3/2_ and Ag 3*d*_5/2_, respectively.^[^ ^25^ ^]^ Hence, the oxidation state of Ag is +1. The fabrication process produces more robust bonding between Ag_44_ NCs and CdS surface than Ag NPs. The Cd 3*d*_3/2_ and Cd 3*d*_5/2_ peaks of APS and ACS‐2 exhibit shifts to low binding energy of 0.04 eV and 0.55 eV, respectively, in comparison with CdS, as shown in Figure 2e, attributable to the formation of a covalent bond between Ag and S atoms and a higher electron cloud density of Cd^2+^.^[^ ^26^, ^27^ ^]^ As shown in Figure 2f, the two peaks of S 2*p* correspond to S 2*p*_1/2_ and S 2*p*_3/2_, respectively.^[^ ^28^ ^]^ In the S 2p spectrum, APS exhibits a shift of 0.09 eV toward lower binding energies, while ACS‐2 demonstrates a more pronounced shift toward lower binding energies. Furthermore, ACS‐2 exhibits a satellite peak at 158.65 eV, which is ascribed to the Ag—S bond formed by the remarkable bonding force between Ag and S. This is also the reason for the significant shifts of the Cd 3*d* and S 2*p* peak.^[^ ^26^ ^]^

The UV‐vis diffuse reflectance spectra (UV‐vis DRS, Figure 2g) reveal that with more Ag_44_ NCs deposition, the light absorption of CdS at the wavelengths of 520–800 nm increases, similar to the light absorption characteristics of Ag_44_ NCs.^[^ ^14^, ^17^ ^]^ No surface plasmon resonance peaks analogous to APS are observed in the 480–520 nm range (Figure S9, Supporting Information), indicating that the Ag_44_ NCs are homogeneously dispersed on CdS without agglomeration.^[^ ^29^ ^]^ The light absorption cut‐off edge of CdS shows a jump of 2.42 eV. In order to analyze the band structure of CdS, ultraviolet photoelectron spectroscopy (UPS) is performed. As shown in Figure 2h, the cut‐off edge of the UPS of CdS is determined to be 17.82 eV, and the work function (*φ*) of CdS is 3.40 eV based on the equation: *φ* = *hv* − E_C_ (*hv* = 21.22 eV). By combining this and the XPS valance band (XPS‐VB, Figure 2i), the valance band maximum (VBM) of CdS is −5.03 eV (*vs* Vacuum). Subsequently, the VBM and the conduction band minimum (CBM) are 0.59 V and −1.83 V (*vs* NHE), respectively, according to E(V) = −4.44 − E(eV).^[^ ^30^ ^]^ The band structure of CdS is consistent with the thermodynamic requirements for the reduction of CO_2_ to C_1_ products (Figure 2j).

**Figure 3:**
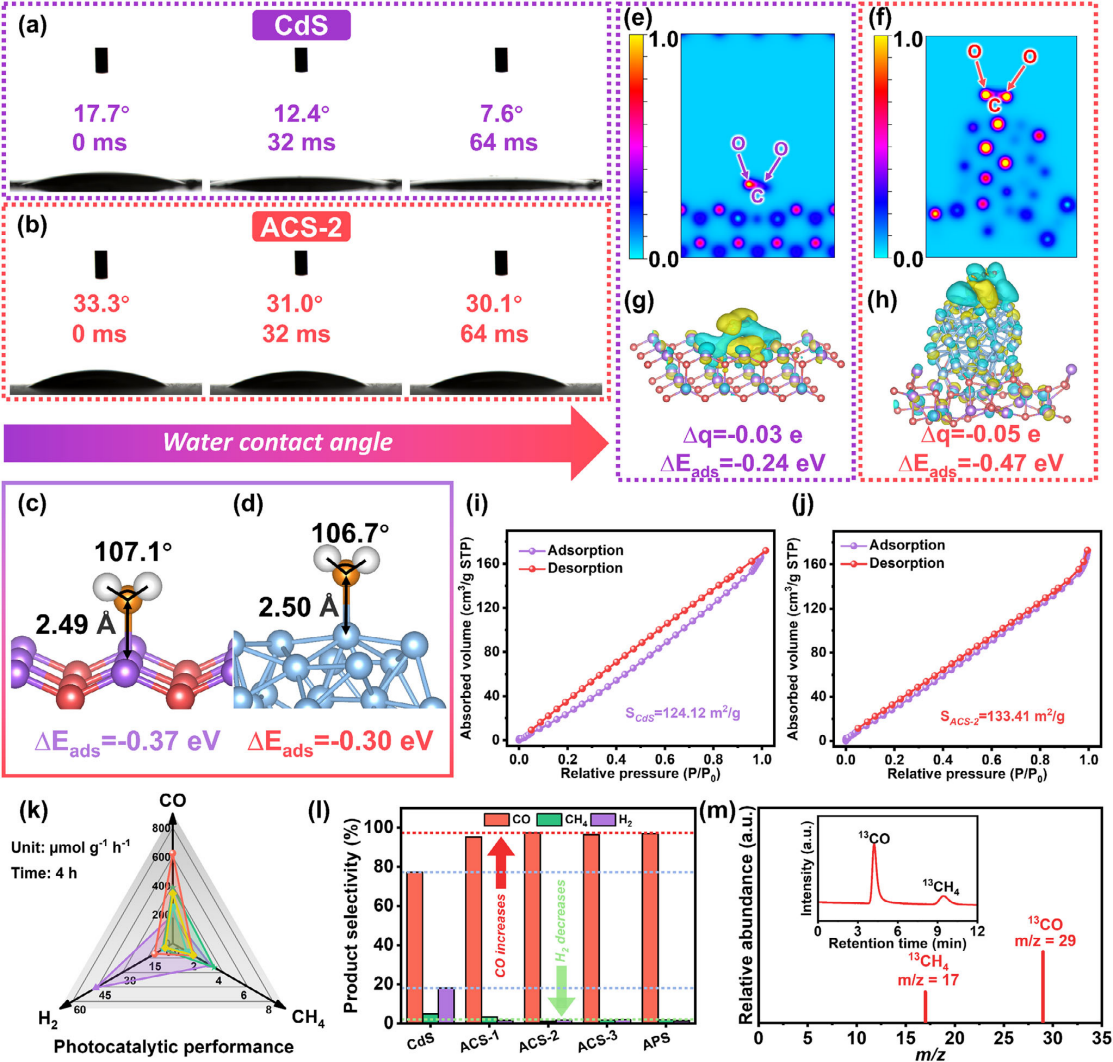
Changes of contact angles with time: a) CdS and b) ACS‐2; Adsorption plots of H_2_O molecules: c) CdS and d) ACS; Local electron functions and charge density differences of CO_2_ adsorbed on: e, g) CdS and f, h) ACS; Physical CO_2_ adsorption and desorption: i) CdS and j) ACS‐2; k) Rates of photocatalytic reduction of CO_2_ products; l) Selectivity of the products of CO_2_ reduction; m) GC‐MS results of the products after photoreduction of ^13^CO_2_ by ACS‐2.

The change in the static water contact angles on CdS and ACS‐2 is monitored. As shown in **Figure** **3**a,b, the water contact angles on ACS‐2 exhibit minimal variation for the same time interval. According to theoretical calculations, the adsorption bond length of ACS‐2 for H_2_O is longer, and the adsorbed H_2_O bond angle is smaller, with a concomitantly smaller adsorption energy relative to CdS (Figure 3c,d). This provides evidence that ACS‐2 has higher hydrophobicity than CdS.^[^ ^23^ ^]^ Subsequently, the electron local functions of CdS and ACS adsorbed with CO_2_ molecules are calculated. As shown in Figure 3e,f, the Ag_44_ NCs function as an electron potential well to facilitate electron accumulation. ACS exhibits a greater capacity for electron transfer to CO_2_ than CdS. As evidenced by the charge density difference and Bader charge calculation (Figure 3g,h), ACS (−0.05 e) transfers 0.02 e more than CdS (−0.03 e). The energies of CO_2_ adsorption on CdS and ACS are −0.24 eV and −0.47 eV, respectively. The deposition of Ag_44_ NCs contributes to the adsorption of CO_2_ onto the catalyst as well as C═O cracking. The physical adsorption properties of the materials for CO_2_ were evaluated. The physical adsorption coefficients (Figure 3i,j) of CO_2_ on CdS and ACS‐2 are 124.12 m^2^ g^−1^ and 133.41 m^2^ g^−1^, respectively. Therefore, the adsorption of CO_2_ by ACS‐2 is more dominant, which is consistent with the calculated results.

The photocatalytic reduction of CO_2_ is evaluated by irradiating with a 300 W Xenon lamp in acetonitrile with H_2_O as the proton source and triethanolamine as the sacrificial agent. After illumination for four hours, ACS‐2 (624.65 µmol g^−1^ h^−1^) exhibits a 2.17‐fold increase in the capacity to activate CO_2_ to CO compared to CdS (197.02 µmol g^−1^ h^−1^, Figure 3k). The CO_2_ reduction performance of ACS‐2 demonstrates a marked improvement over other similar works, rendering it more competitive (Table S2, Supporting Information). However, when the loading of Ag_44_ NCs exceeds 3.36 wt% (Table S3, Supporting Information), the catalytic properties degrade on account of the “shielding effect” by an excessive amount of Ag_44_ NCs.^[^ ^31^ ^]^ APS (3.32 wt%), with the same Ag loading as ACS‐2 (3.36 wt%), reduces CO_2_ to CO at a rate of 345.47 µmol g^−1^ h^−1^. However, the enhancement is not as pronounced as that of ACS‐2. This is because the ultrasmall Ag_44_ NCs reduce the electron transport distance and enhance electron aggregation at the active sites. The introduction of Ag led to the formation of shuttling channels for directional migration of electrons to CO_2_,^[^ ^32^, ^33^ ^]^ consequently reducing the coupling between electrons and H^+^ and inhibiting HER. As shown in Figure 3l, the introduction of both Ag_44_ NCs and Ag NPs resulted in a notable decline in the H_2_ selectivity, from 18% to below 2%. In particular, ACS‐2 exhibits near 100% selectivity in the conversion of CO_2_ to CO.

The CO_2_ activation properties in a series of reactions are evaluated. Under dark conditions, the activation of CO_2_ is precluded since the catalyst is unable to absorb light to excite electrons. After switching on the Xenon lamp, the yield of CO_2_ reduction increases with the duration of illumination (Figure S10a, Supporting Information). As shown in Figure S10b (Supporting Information), no gas product is formed in the absence of ACS‐2, suggesting that the photocatalyst is necessary for the photocatalytic reduction of CO_2_. Throughout the reduction process, only H_2_ is produced when CO_2_ is replaced with Ar. It is hypothesized that the C source of CO and CH_4_ is produced by the reduction of CO_2_ in the activation process. To exclude the possibility that CO and CH_4_ are produced by the decomposition of adsorbed carbon, acetonitrile and triethanolamine are used on the catalyst surface, and ^13^C tracing experiments are conducted. As shown in Figure 3m, the fragments with m/z = 17 and 29 detected by GC‐MS are ^13^CH_4_ and ^13^CO, respectively. This demonstrates that the ^13^C source is ^13^CO_2_, and it provides compelling evidence that the gaseous C product is derived from CO_2_. After the completion of the stability testing of ACS‐2 and following three cycles, the yield of CO declines slightly (Figure S10c, Supporting Information). The catalyst displays a viscous appearance on the surface due to the photodegradation of CdS after prolonged high‐power irradiation by the Xenon lamp. The ACS‐2 structure is examined microscopically. As shown in Figure S11a (Supporting Information), EDS does not detect other elements besides Ag, S, and Cd, and CdS retains the rod‐like morphology (Figure S11b, Supporting Information). Figure S11c‐f (Supporting Information) reveals that the distributions of Cd, S, and Ag, and the Ag_44_ NCs are firmly anchored on CdS without detachment.

**Figure 4:**
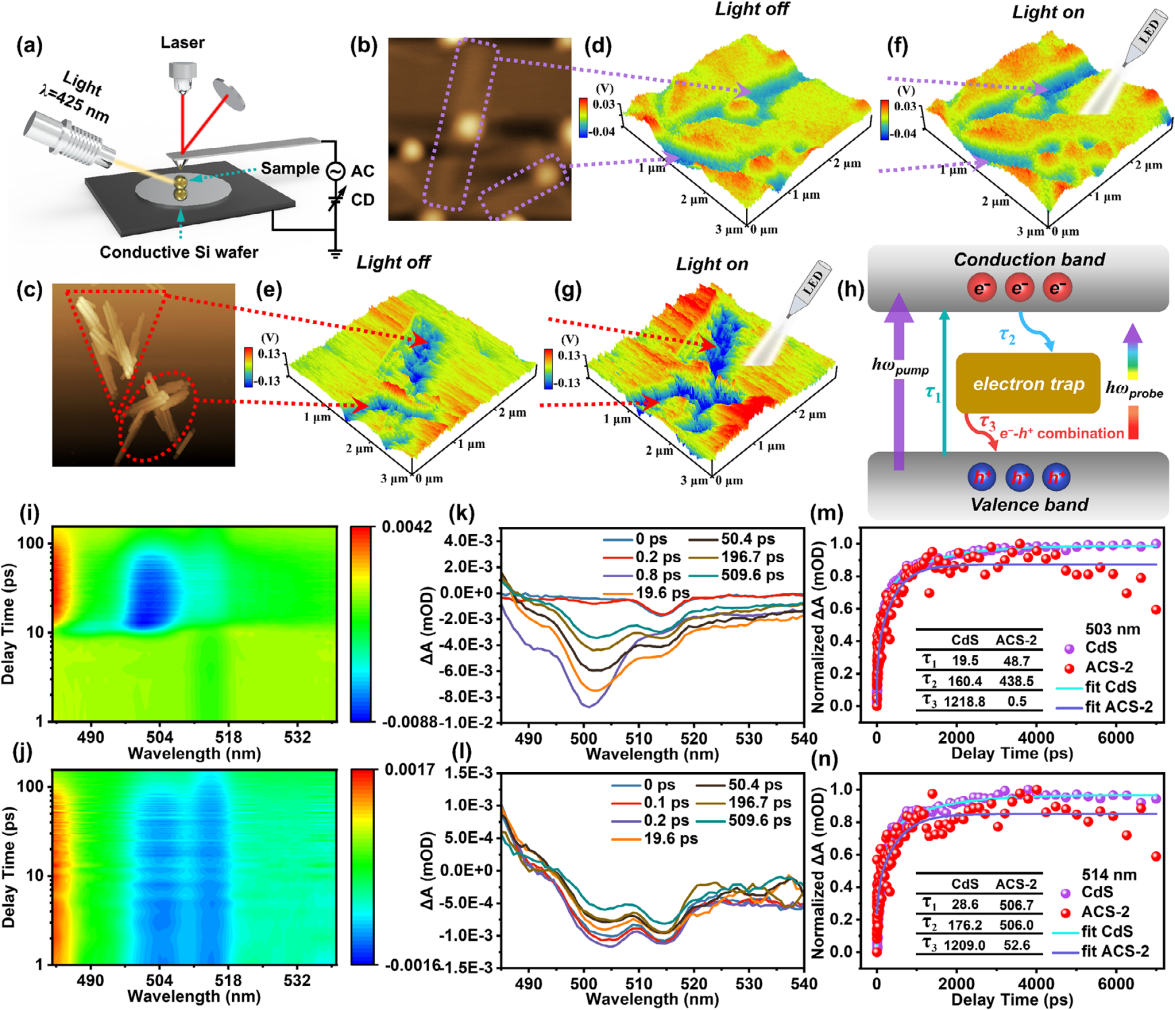
a) Schematic diagram of the KPFM measurement; AFM images of b) CdS and c) ACS‐2; Surface potential maps before illumination of d) CdS and e) ACS‐2; Surface potential maps after illumination of f) CdS and g) ACS‐2; h) Schematic diagram of the electronic relaxation process; 2D TAS of i) CdS and j) ACS‐2; TA signal spectra of k) CdS and l) ACS‐2 on the fs–ps timescale; Normalized TAS decay kinetics curves at m) 503 nm and n) 514 nm of CdS and ACS‐2.

The surface potentials are evaluated by Kelvin probe force microscopy (KPFM, **Figure** **4**a). After performing atomic force microscopy (AFM, Figure 4b,c). Subsequently, the surface potentials are measured from selected areas. In comparison with CdS (Figure 4d), ACS‐2 (Figure 4e) shows a larger surface potential difference from 0.07 V to 0.26 V. Meanwhile, under illumination by a 425 nm LED lamp, the surface potential difference of ACS‐2 (Figure 4f) fluctuates more than that of CdS (Figure 4g). The Ag_44_ NCs facilitate the excitation and aggregation of a significant number of photogenerated electrons.^[^ ^34^, ^35^ ^]^ The surface photovoltage (SPV) reflects the electronic properties, specifically the charge separation efficiency.^[^ ^36^ ^]^ The SPV (Figure S12, Supporting Information) of ACS‐2 (0.022 mV) increases by 0.017 mV relative to CdS (0.005 mV) under photoexcitation in the range of 300–525 nm, indicating that ACS‐2 is more efficient in charge separation due to polarization provided by Ag_44_ NCs.

The incident photon‐to‐electron conversion efficiency (IPCE) determines the efficiency with which incident photons are converted to electrons. The IPCE (Figure S13, Supporting Information) of ACS‐2 is consistently higher than that of CdS for the same excitation wavelength. These findings indicate that the molecule‐like properties of Ag_44_ NCs enable them to act as photosensitizers to enhance the visible light utilization efficiency.^[^ ^37^ ^]^ As shown in Figure S14 (Supporting Information), the photoelectric signal of ACS‐2 is greater, and photogenerated electron excitation is stronger.^[^ ^38^ ^]^ As shown by electrochemical impedance spectroscopy (EIS, Figure S15, Supporting Information), the Nyquist radius of ACS‐2 is smaller than that of CdS, suggesting that ACS‐2 has better efficiency in separating electron‐hole pairs.^[^ ^39^, ^40^ ^]^ Figure S16 (Supporting Information) displays the steady‐state photoluminescence spectra at an excitation wavelength of 400 nm. ACS‐2 exhibits dramatic PL quenching, indicating the presence of an electron transfer channel for Cd‐S‐Ag and subsequently accelerated transfer of photogenerated electrons.^[^ ^41^ ^]^ The average fluorescence lifetime (τ_ave_) of ACS‐2 (2.46 ns) is less than that of CdS (3.38 ns), as shown by the time‐resolved PL (TRPL, Figure S17 and Table S4, Supporting Information) spectra following a second‐order exponential fit, which shows a larger charge transfer rate constant (k = 1/τ_ave_) for ACS‐2. This results in the notable enhanced carrier separation kinetics of ACS‐2.^[^ ^42^, ^43^ ^]^

Femtosecond time‐resolved transient absorption spectroscopy (fs‐TAS) is performed on CdS and ACS‐2,^[^ ^44^ ^]^ and the photocatalytic mechanism is illustrated in Figure 4h. When the catalyst is photoexcited, photoelectrons are excited from the ground state and relax to the conduction band (τ_1_), corresponding to the generation of the broad photoinduced absorption signal. Subsequently, photoelectrons undergo relaxation from the conduction band to an intermediate trap (τ_2_). Thereafter, the photoelectrons transfer from the trap to the ground state, where they recombine with holes (τ_3_) (average lifetime within the instrument response function of the fs pump−probe spectrometer).^[^ ^45^ ^]^ As shown in Figure 4i and j, the 2D TAS reveals that both CdS and ACS‐2 exhibit distinct positive absorption peaks at 482–486 nm from the excited state absorption (ESA) signals generated by a 400 nm pump pulse.^[^ ^41^ ^]^ The two negative ground state bleaching (GSB) signals at 503 nm and 514 nm are the relaxation signals of the excited states of CdS (Figure 4k) and ACS‐2 (Figure 4l).^[^ ^46^ ^]^ In the GSB signal, a notable recovery of the two ACS‐2 peaks is observed in comparison with CdS. A third‐order nonlinear fitting is performed on the GSB signal, while third‐order nonlinear fitting is conducted on the GSB signal, with τ_1_, τ_2_, and τ_3_ corresponding to the lifetimes of carrier relaxation, electron transfer to the trap, and electron recombination from the trap to the ground state, respectively.^[^ ^47^, ^48^, ^49^ ^]^ At a wavelength of 503 nm (Figure 4m and Table S5, Supporting Information), the lifetime of ACS‐2 (τ_1_ = 48.7 ps, A_1_ = 3.3%, τ_2_ = 438.5 ps, A_2_ = 96.0%) is longer than that of CdS (τ_1_ = 19.5 ps, A_1_ = 0.7%, τ_2_ = 160.4 ps, A_2_ = 18.6%). Ag_44_ NCs have been shown to generate a greater number of excited electrons on the CdS surface,^[^ ^50^ ^]^ with a significant proportion of these excited electrons evading both bulk and surface recombination processes, and transferring to the Ag_44_ NCs potential well. Meanwhile, the τ_3_ lifetime and A_3_ value of ACS‐2 were found to be close to zero, indicating that only a negligible fraction of electrons captured by the silver potential well would return to the CdS surface for recombination. The most notable enhancement in the τ_1_ lifetime of ACS‐2 (Figure 4n) is observed at a wavelength of 514 nm. Hence, Ag_44_ NCs have high absorbance at this wavelength, leading to substantial electronic excitation on CdS (Table S6, Supporting Information, CdS: τ_1_ = 28.6 ps, A_1_ = 0.8%; ACS‐2: τ_1_ = 506.7 ps, A_1_ = 48.7%). The τ_3_ lifetime and A_3_ of ACS‐2 are less than those of CdS, resulting in fewer electron‐hole recombination. This detailed charge transfer dynamics demonstrates the existence of an electron shuttling channels at the Ag_44_ NCs/CdS interface. The average charge lifetimes at the two wavelengths are calculated (Tables S5 and S6, Supporting Information). The average charge lifetime of ACS‐2 is slightly shorter than that of CdS. This finding corroborates that the charge transfer rate of ACS‐2 is faster,^[^ ^47^, ^51^ ^]^ a conclusion consistent with TRPL.

**Figure 5:**
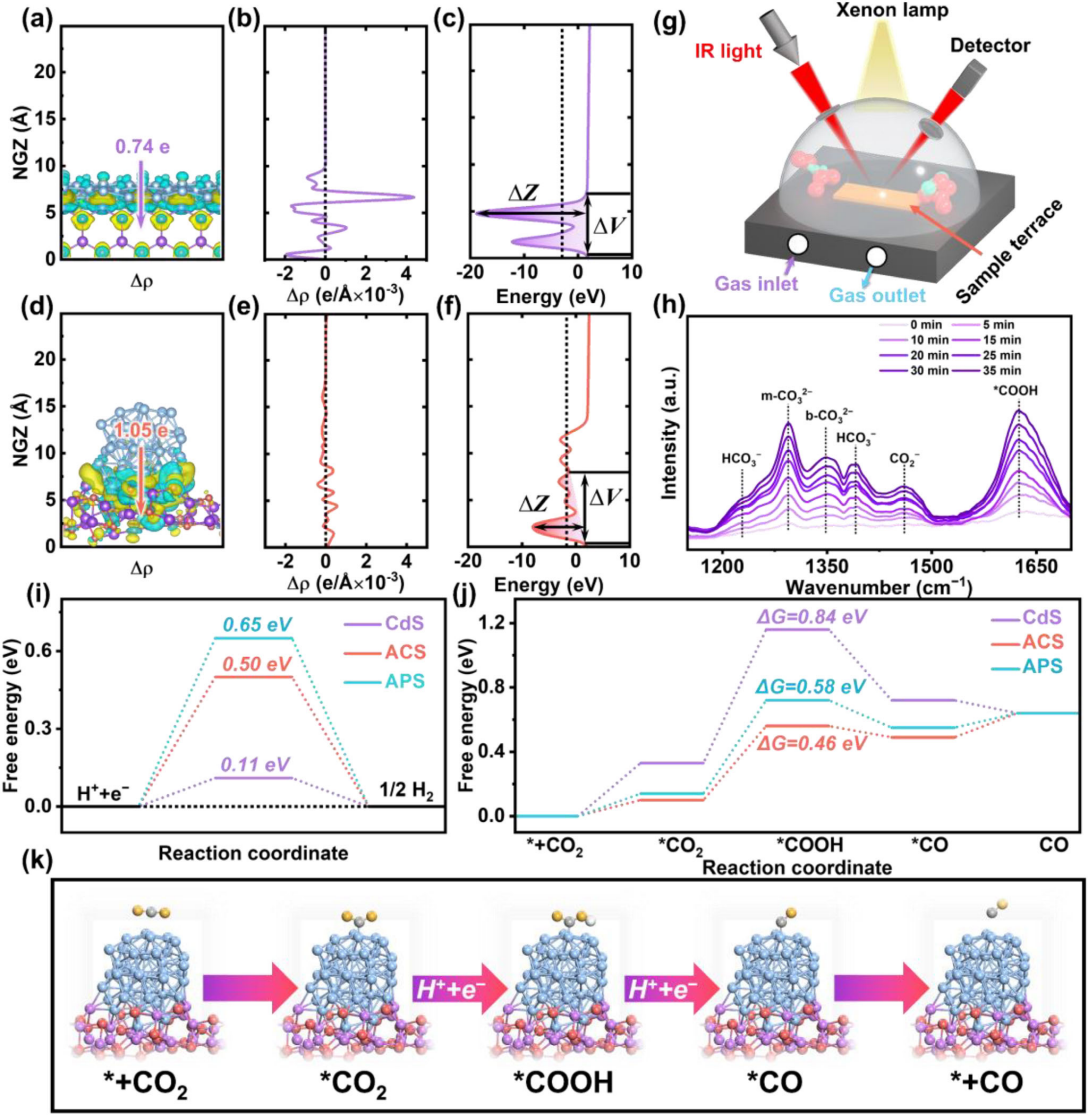
Electronic structure changes of a‐c) APS and d‐f) ACS: (a, d) Calculated differential charge, (b, e) Plane‐averaged charge density difference, (c, f) Plane‐averaged electrostatic potential along the Z direction; g) Schematic diagram of the in situ FTIR apparatus; h) In situ FTIR spectra of ACS‐2; i) Gibbs free energy for hydrogen production on CdS, ACS, and APS; j) Free energy of activated CO_2_ to CO on CdS, ACS, and APS; k) Activation of CO_2_ to CO by ACS.

In order to elucidate the mechanism by which ACS achieves highly selective reduction of CO_2_ to CO while inhibiting the hydrogen evolution reaction, theoretical calculations and in situ Fourier Transform infrared spectroscopy (FTIR) are conducted. As shown in **Figure** **5**a,d, the differential charge density exhibits a notable redistribution of charge densities in the vicinity of the ACS interface. In order to quantify electron transfer, the Bader charge (∆q) shows that the charges transferred from CdS to Ag NPs and Ag_44_ NCs are 0.74 e and 1.05 e, respectively. The plane‐average charge density difference of ACS fluctuates less than that of APS, as shown in Figure 5b,e. The number of electrons for each Ag atom on Ag_44_ NCs is similar, and each Ag atom is fully utilized. This allows Ag_44_ NCs to act as an electron potential well to retain electrons and reduce of electron leap to the ground state. The plane‐averaged electrostatic potentials along the Z‐direction of the APS and ACS are calculated to investigate the interfacial charge transfer in the presence of the potential energy barrier. As shown in Figure 5c,f, there is a considerable tunnelling barrier at the Fermi level for APS with ∆V = 20.60 eV and ∆Z = 6.57 Å. This impedes the transfer of electrons at the interface. In contrast, ∆V = 9.80 eV and ∆Z = 7.90 Å for ACS, thus diminishing the tunneling barrier at the Fermi level and showing that electrons at the Fermi level can readily transfer along the Ag_44_ NCs barrier.^[^ ^52^ ^]^ In situ FTIR (Figure 5g) is carried out to simulate the CO_2_ reduction environment (1D in situ FTIR (Figure 5h) and 2D in situ FTIR (Figure S18, Supporting Information)). The peaks at 1228 cm^−1^ and 1392 cm^−1^ arise from HCO_3_^−^, and those at 1294 cm^−1^ and 1348 cm^−1^ stem from m‐CO_3_^−^ and b‐CO_3_^−^, respectively. The peaks at 1461 cm^−1^ and 1625 cm^−1^ are attributed to CO_2_^−^ and *COOH, respectively, which serve as pivotal intermediates in the reduction of CO_2_ to CO.^[^ ^42^, ^53^ ^]^ The peak intensity of the intermediates increases with illumination in accordance with the product yields over time. The intermediates in the reduction of CO_2_ to methane or C_2_ products, such as *CHO and *HCOOH, are not observed by in situ FTIR.^[^ ^54^ ^]^

The intermediates associated with hydrogen production by hydrolysis cannot be identified by in situ FTIR spectroscopy alone. Accordingly, in order to account for the abated hydrogen production by Ag, the Gibbs free energy for hydrogen production is calculated. As shown in Figure 5i, after the deposition of Ag NPs and Ag_44_ NCs on CdS, the hydrogen production ∆G values of CdS, APS, and ACS are 0.11, 0.65, and 0.50 eV, respectively, because of the energy barriers impeding hydrogen production. A model is constructed for the reduction of CO_2_ to CO to calculate the activation free energy (Figure s19‐s21, Supporting Information). The results indicate that the energy barrier for the rate‐determining step of ACS activation of CO_2_ is the lowest (Figure 5j,k, Table S7‐S9, Supporting Information). Consequently, the *CO intermediate favors direct desorption and cannot provide the energy required for the subsequent reaction of *CO to produce CH_4_. The experimental and computational results demonstrate that Ag_44_ NCs function as active sites, facilitate electron aggregation, and inhibit the combination of electrons with H^+^ to generate hydrogen. This results in a CO selectivity of 97.3%.

## 3 Conclusion

Ag_44_ NCs are anchored onto CdS by a low‐temperature electrostatic deposition method. The Ag_44_ NCs form an electron potential well on the CdS surface and long‐range ordered electric fields for directional electron transfer. Positively charged Ag_44_ NCs inhibit the combination of electrons and protons to produce hydrogen under electrostatic repulsion. Concurrently, the molecule‐like characteristics of Ag_44_ NCs facilitate CO_2_ adsorption, while the surface geometry increases the number of active sites on the materials. In particular, ACS‐2 has abundant active sites, enhanced directional electron transfer, and a low activation energy barrier. The efficiency of the photocatalytic reduction of CO_2_ improves while hydrogen evolution is inhibited, resulting in a CO_2_‐to‐CO yield that is three times higher than that of CdS. The CO selectivity is 97.3% and that of H_2_ is 1.7%. The results reveal an innovative framework and provide insights into the use of metal nanoclusters for photocatalytic CO_2_ reduction.

## 4 Experimental Section

### Chemicals

The main chemicals included cadmium nitrate tetrahydrate (Cd(NO_3_)_2_·4H_2_O, ≥99.0%), thiourea (CH_4_N_2_S, ≥99.0%), ethylenediamine (C_2_H_8_N_2_, ≥99.0%), *para*‐mercaptobenzoic acid (p‐MBA, 90.0%), sodium borohydride (NaBH_4_, AR), cesium hydroxide monohydrate (CsOH·H_2_O, 95.0%), N,N‐dimethylformamide (DMF, ≥99.5%), silver nitrate (AgNO_3_, ≥99.8%), acetic acid (HOAc, ≥99.5%), and ethanol. All of the chemicals were analytical grade and used without further treatment. Ultrapure water (18.25 MΩ·cm) was used in the experiments.

### Synthesis of Ag_44_ NCs

Silver nitrate (21.25 mg) was dissolved in 5.25 mL of water, and p‐MBA (38.40 mg) was dissolved in 3 mL of ethanol. The two solutions were mixed in a 25 mL glass vial to form a yellow suspension, and the pH was adjusted to 12 with 50 wt% aqueous CsOH. At this pH, the solution became transparent. Subsequently, 2.25 mL of a freshly prepared sodium borohydride solution (23.67 mg NaBH_4_ dissolved in 2.25 mL of 0.1 M CsOH solution) were added and heated in a water bath to 50 °C under vigorous stirring for two hours. The color changed from clear to dark purple. The suspension was centrifuged at 10000 rpm for 5 min. The supernatant was combined with 10 mL of ethanol and centrifuged again at 10000 rpm for 5 min. The precipitate was then collected, followed by the addition of 5 mL of HOAc:DMF (1:9). Toluene (10 mL) was added. The protonated Ag_44_ NCs precipitate was collected after centrifugation at 10000 rpm for 5 min and dissolved in DMF for further use.

### Synthesis of CdS Nanorods

Cd(NO_3_)_2_·4H_2_O (5 g) and CH_4_N_2_S (3.7 g) were placed in 60 mL of C_2_H_8_N_2_ and stirred for about 40 min, after which it was transferred to a reactor and heated to 180 °C for 24 h. The precipitate was collected after centrifuging and washing several times with anhydrous ethanol and deionized water. The CdS samples were vacuum‐dried at 60 °C for 8 h.

### Synthesis of Ag_44_ NCs/CdS

CdS (100 mg) was dispersed in a beaker containing 20 mL of water, and 0.05 mL, 0.3 mL, and 0.6 mL of Ag_44_ NCs were added, respectively. The mixture was sonicated for 20 min, and a small amount of liquid nitrogen was added to reduce the temperature. Finally, liquid nitrogen was added to freeze it prior to vacuum freeze‐drying. The samples were designated ACS‐1, ACS‐2, and ACS‐3, respectively.

## Conflict of Interest

The authors declare no conflict of interest.

## Author Contributions

H.X. wrote – original draft, conceptualization, investigation, data curation, methodology. X.L. methodology, wrote – review and editing. G.Z. methodology. C.B. wrote – review and editing. Q.L. methodology. W.J. methodology, wrote – review and editing. B.W. methodology, wrote – review & editing. X.Z. wrote – review & editing, funding acquisition, conceptualization. P.K.C. resources, methodology, wrote – review and editing. X.W. resources, funding acquisition, methodology, writing – review and editing.

## Supporting information



Supporting Information

## Data Availability

The data that support the findings of this study are available from the corresponding author upon reasonable request.
