# Supplementary material for: Controlled Photocatalytic Reduction of CO2 by Precise Atomic‐Level Interface Modification and Engineering of Silver Nanoclusters
Source: Adv Sci (Weinh). 2025 Oct 24;13(1):e16096. doi: 10.1002/advs.202516096 (PMC12767042; doi:10.1002/advs.202516096)
Supplement: Supplementary file 1 — Supporting Information [file ADVS-13-e16096-s001.pdf]

Supporting Information

Controlled Photocatalytic Reduction of CO_2 by Precise Atomic-Level Interface Modification and Engineering of Silver Nanoclusters

Hangmin Xu[a], Xiang Liu[a], Ganghua Zhou[a], Chuanzhou Bi[a], Qing Liu[a], Weiyi Jiang[a], Bin Wang[b], Xingwang Zhu[a], Paul K. Chu[b*], Xiaozhi Wang[a*]*

[a] School of Mechanical Engineering, College of Environmental Science and Engineering, Yangzhou University, Yangzhou 225009, P. R. China

[b] Department of Physics, Department of Materials Science and Engineering, and Department of Biomedical Engineering, City University of Hong Kong, Kowloon 999077, Hong Kong, China

* Corresponding authors: zxw@yzu.edu.cn (X. Zhu); paul.chu@cityu.edu.hk (P.K. Chu); xzwang@yzu.edu.cn (X. Wang)

Materials characterization

The crystal structure of the photocatalysts was determined by X-ray diffraction (XRD, D8 Advance, Bruker AXS, Germany), and the chemical states were analyzed by X-ray photoelectron spectroscopy (XPS, ESCALABC 250Xi, Thermo Scientific, USA). Field-emission scanning electron microscopy (SEM, S-4800II, Japan), transmission electron microscopy (TEM, Tecnai 12, Philips), and high-resolution transmission electron microscopy (HR-TEM, Tecnai G2 F30 S-TWIN, USA) were performed to observe the morphology of the materials. The optical properties were determined by UV-vis diffuse reflectance spectroscopy (Cary 5000, Varian, USA), and a surface area analyzer (ASAP2460, Micromeritics Instrument Co., USA) was used to determine the N_2 adsorption-desorption isotherms of the catalysts. The surface topography was observed by atomic force microscopy (AFM, SPM-9700HT, Shimadzu, Japan). The steady-state fluorescence spectra were acquired (Edinburgh, FLS980), while the steady-state surface photovoltages (SPV) and incident photon-to-current efficiency (IPCE) were measured on the PL-SPV/IPCE1000 (PerfectLight, China). The Cu concentration was determined by inductively coupled plasma mass spectrometry (ICP-MS, iCAPTM QC, USA), and the *in situ* FTIR spectra were obtained on the Thermo Scientific Nicolet iS50.

Photo/electrochemical assessment

The photo/electrochemical properties of the catalysts were determined using an electrochemical workstation (DH7000C, Donghua Instrument Co., Ltd., China) with the three-electrode configuration comprising a Pt wire electrode as the reference electrode, an Ag/AgCl electrode as the counter electrode, and ITO conductive glass as the working

electrode. The working electrode was prepared by dispersing 5 mg of the photocatalyst in 950 μL of ethanol and 50 μL of Nafion. The suspension (70 μL) was placed on the 1 × 1 cm ITO conductive glass. Using 0.2 M Na_2SO_4 as the electrolyte and a 300 W Xe lamp as the light source, the transient photocurrents were measured, and electrochemical impedance spectroscopy (EIS) was carried out.

Photocatalytic activity

Ten mg of the catalyst, 6 mL of acetonitrile, 4 mL of H_2O, and 2 mL of trolamine were mixed ultrasonically. The water-cooled reactor (10 °C) was evacuated to 20 kPa, and then high-purity CO_2 was introduced to reach a pressure of 80 kPa to purge residual gases at 10 °C (water-cooled). A 300 W xenon lamp irradiated the sample under constant agitation. The gas samples were collected from the reactor every hour for gas chromatography. Each test cycle lasted 4 hours.

Theoretical computations

Density-functional theory (DFT) calculations were performed using the Vienna ab-initio simulation package (VASP) for structural optimization. The PBE exchange-correlation functional of the generalized gradient approximation (GGA) was used to describe the exchange-correlation energy. The projection suffix plus plane wave method was adopted to treat the wave functions with the cut-off energy set to 520 eV. The dispersion correction method (DFT-D3) was employed to examine the van der Waals interactions, and a 3 × 3 × 1 Monkhorst-Pack K-point grid was used to sample the K points of the Brillouin zone integrals. The criteria for the optimization of the theoretical structural

model and convergence for the electronic structure calculations were: total energy change < 0.05 eV $Å^{-1}$ and ion relaxation energy $< 10^{-5}$ eV. The ground state structures of CO_2*, COOH*, and CO* adsorbed on the catalyst surface were determined by evaluating all the possible configurations on all active sites and choosing the one with the lowest energy. The free energy for adsorbates and non-adsorbed gas-phase molecules was calculated by the following equation:

$$\Delta G = E_{total} - E_{slab} - E_{mol} + \Delta E_{ZPE} - T\Delta S \,, \qquad (S1)$$

where E_{total} is the total energy of the adsorption state, E_{slab} is the energy of the pure surface, E_{mol} is the energy of an adsorbed molecule, ΔE_{ZPE} is the zero-point energy change, and ΔS is the entropy change.

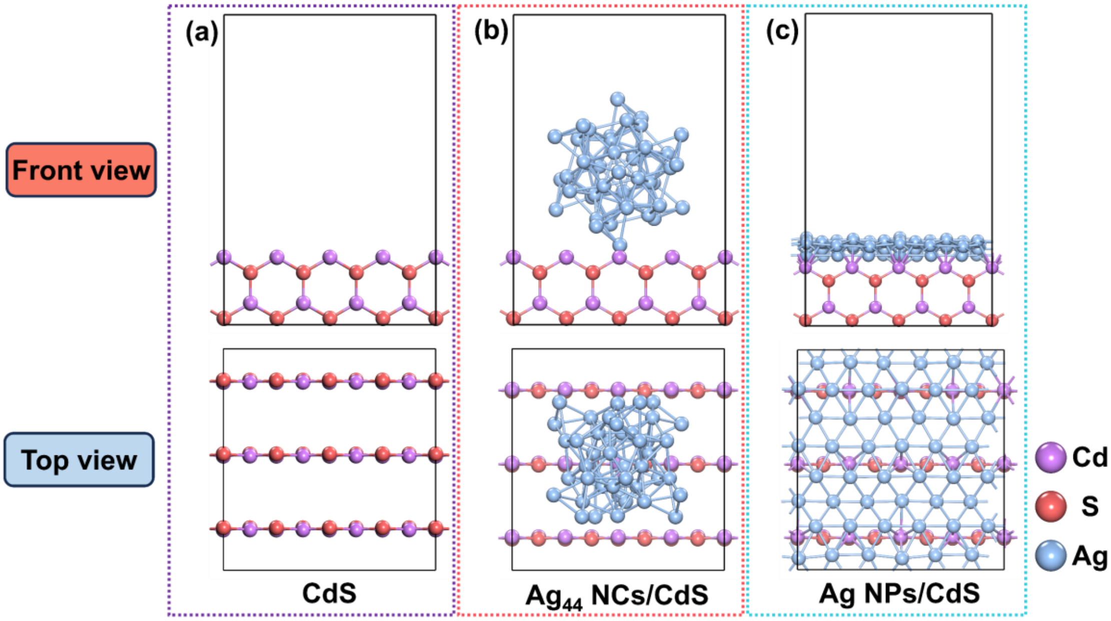

Figure S1. Theoretical calculation models: (a) CdS, (b) ACS, and (c) APS.

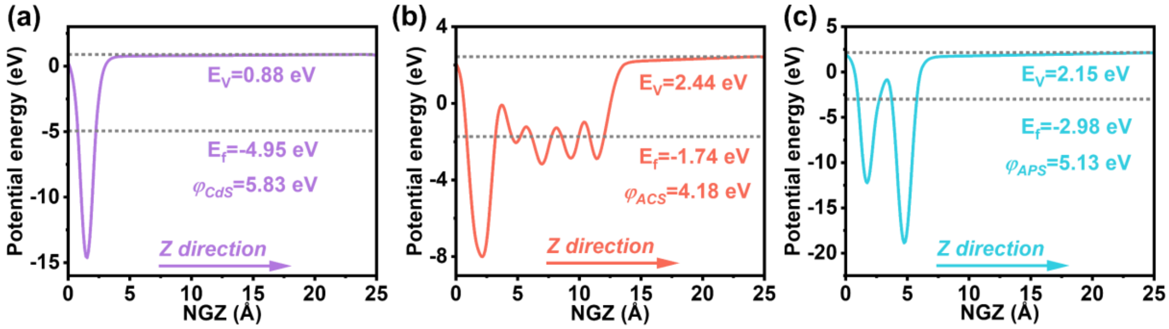

Figure S2. Calculated work functions along the z-axis: (a) CdS, (b) ACS, and (c) APS.

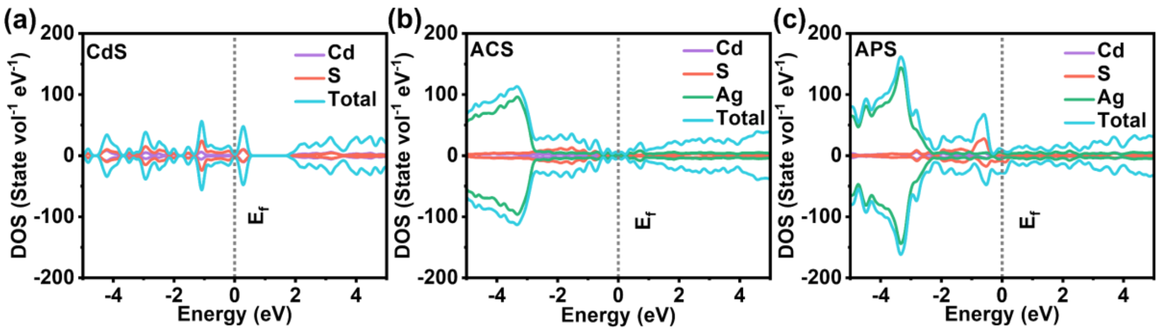

Figure S3. DOS of (a) CdS, (b) ACS, and (c) APS with the dashed lines denoting the Fermi level set at 0 eV.

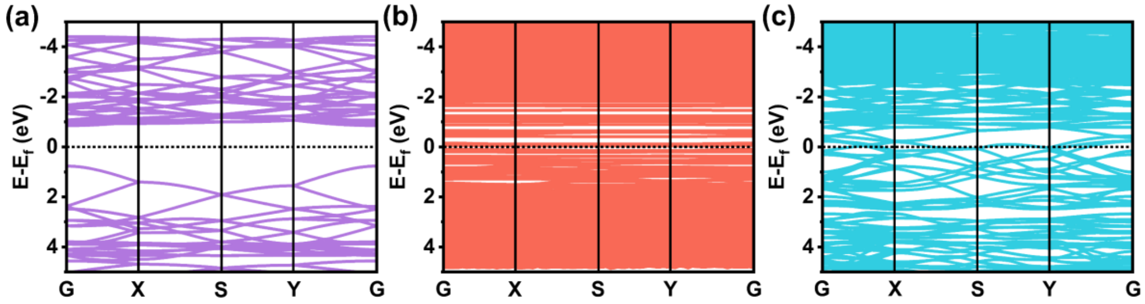

Figure S4. Band structures of (a) CdS, (b) ACS, and (c) APS.

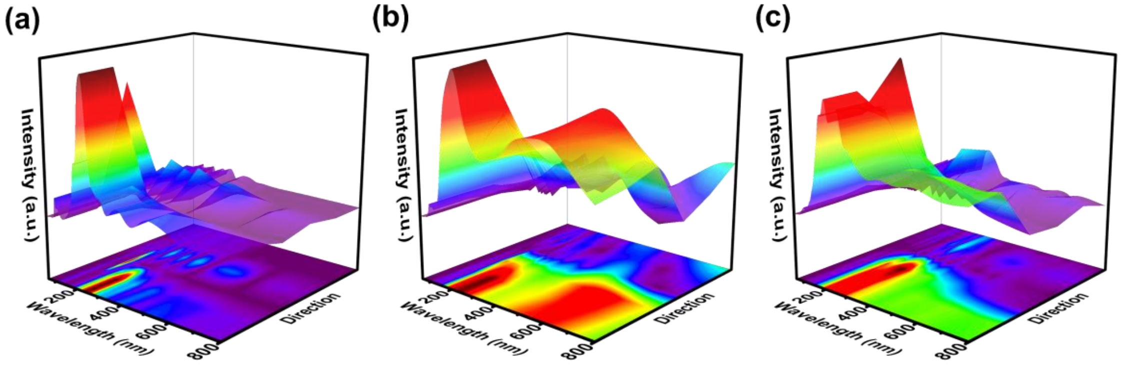

Figure S5. Light absorption spectra of (a) CdS, (b) ACS, and (c) APS.

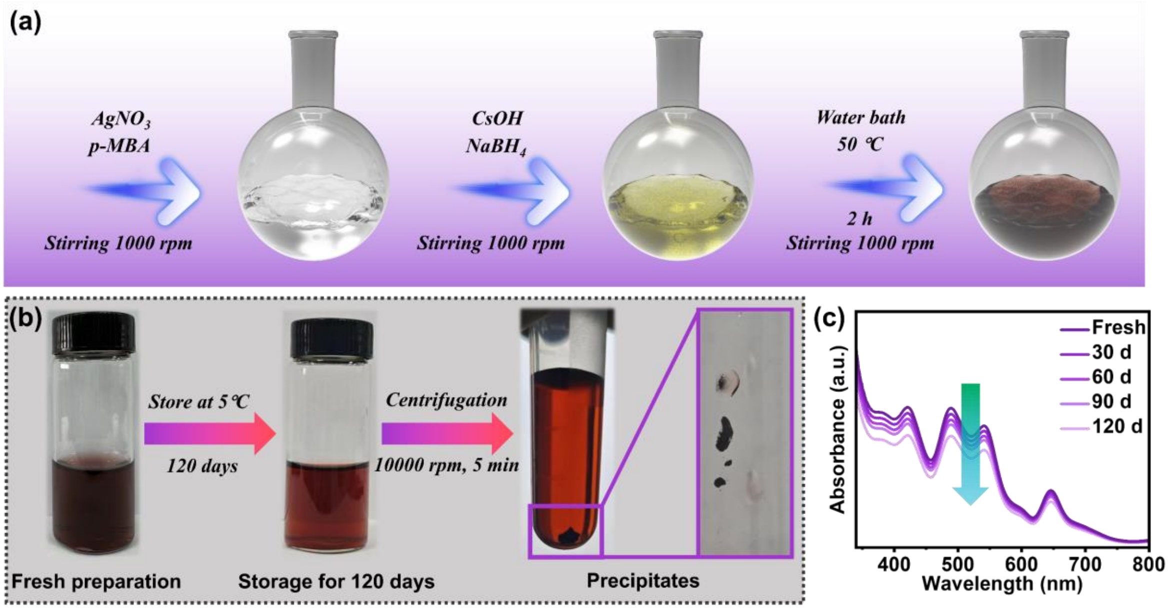

Figure S6. (a) Schematic of the synthesis of Ag_{44} NC, (b) Pictures showing the changes of Ag_{44} NCs without protonation after long-term storage, and (c) Changes of UV absorption spectra of Ag_{44} NCs with storage time.

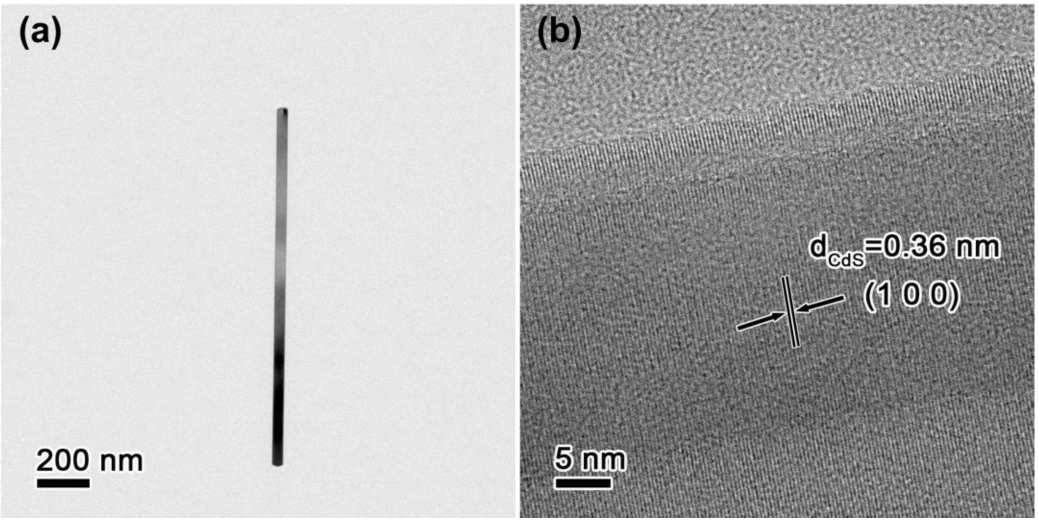

Figure S7. (a) TEM image of ACS-2 and (b) HR-TEM image of CdS.

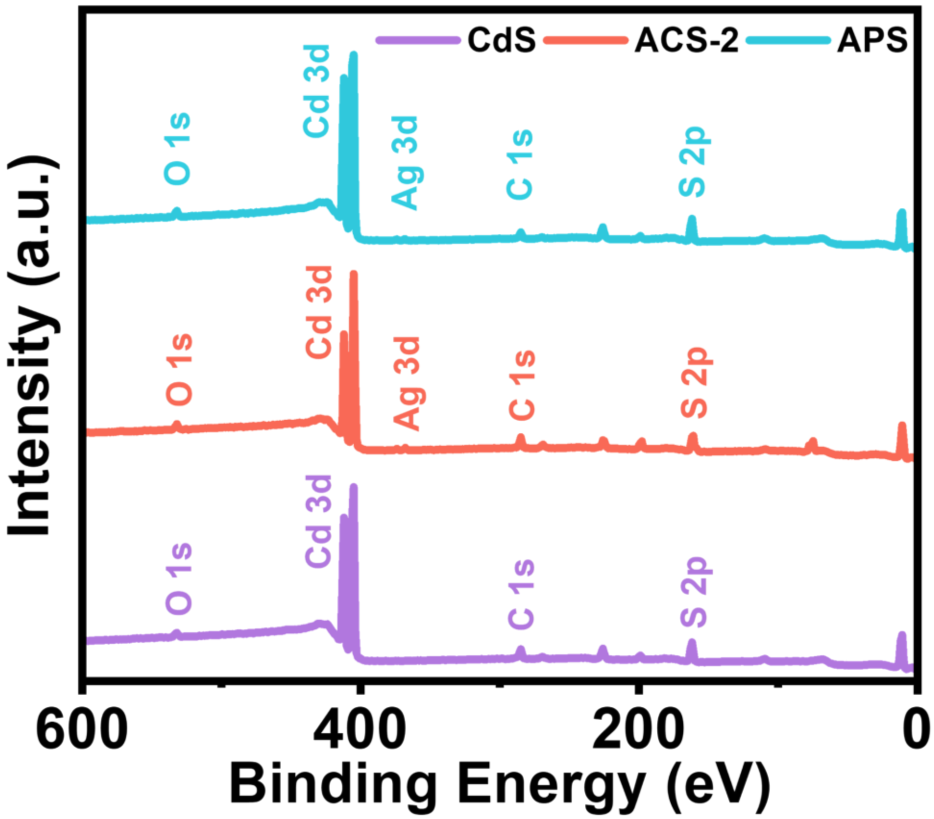

Figure S8. XPS survey spectra of CdS, ACS-2, and APS.

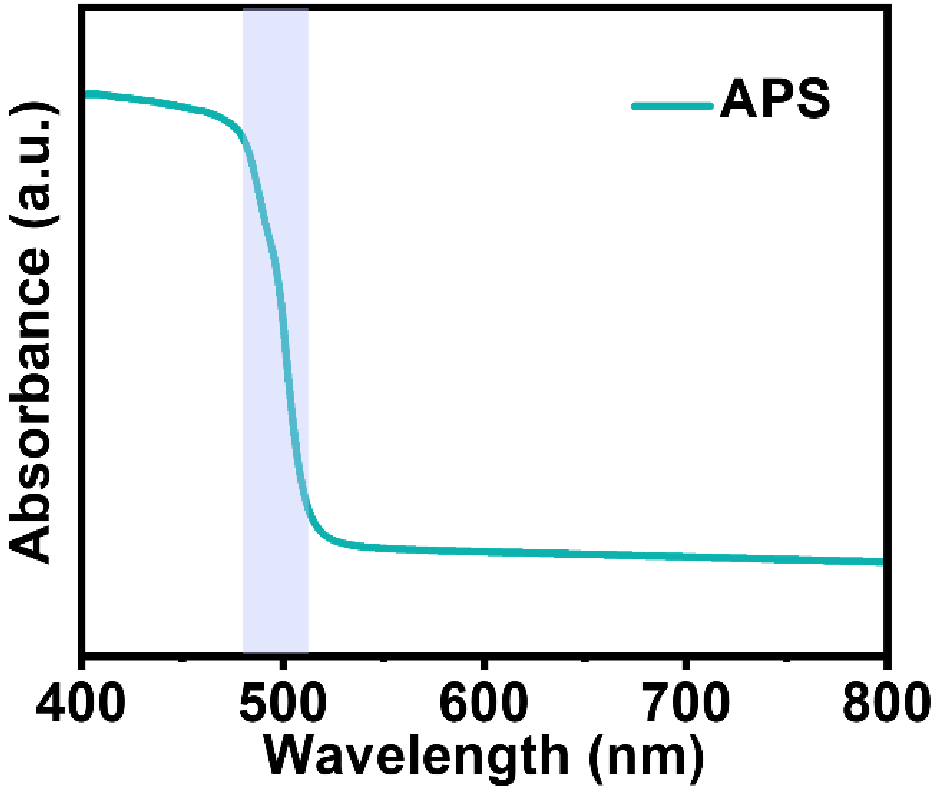

Figure S9. UV-visible diffuse reflectance spectra of APS.

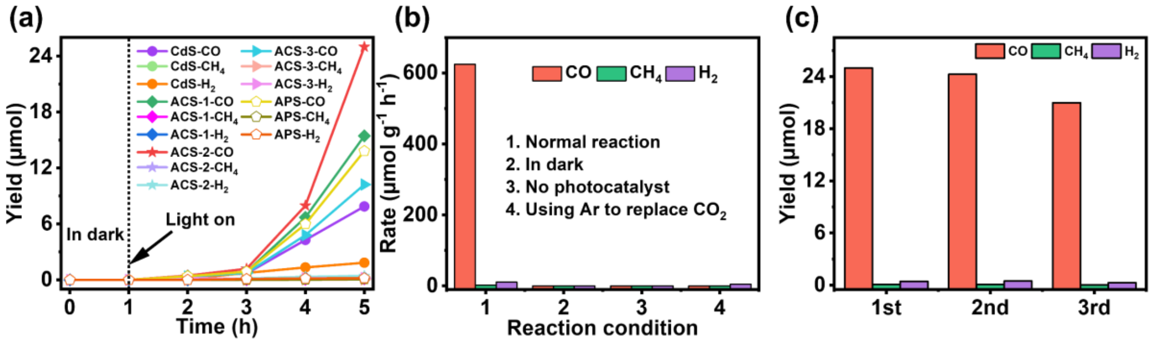

Figure S10. (a) Product yield versus time in the photocatalytic carbon dioxide reduction process, (b) CO_2 reduction of ACS-2 under different conditions, and (c) Cyclic experiments of ACS-2.

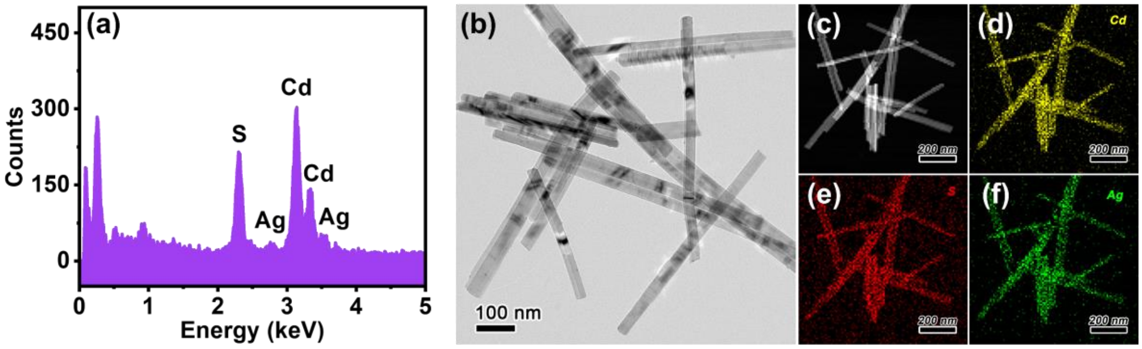

Figure S11. (a) EDS spectrum, (b) HR-TEM image, (c) STEM image, and (d-f) EDS elemental maps of ACS-2 after the reaction.

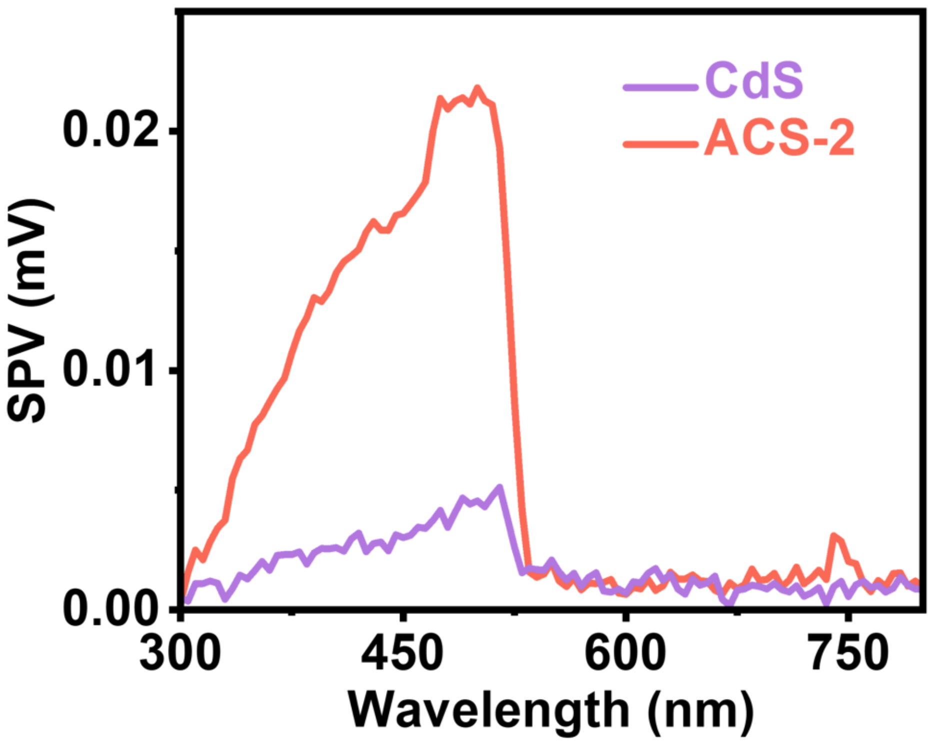

Figure S12. Surface photovoltages (SPV) of CdS and ACS-2.

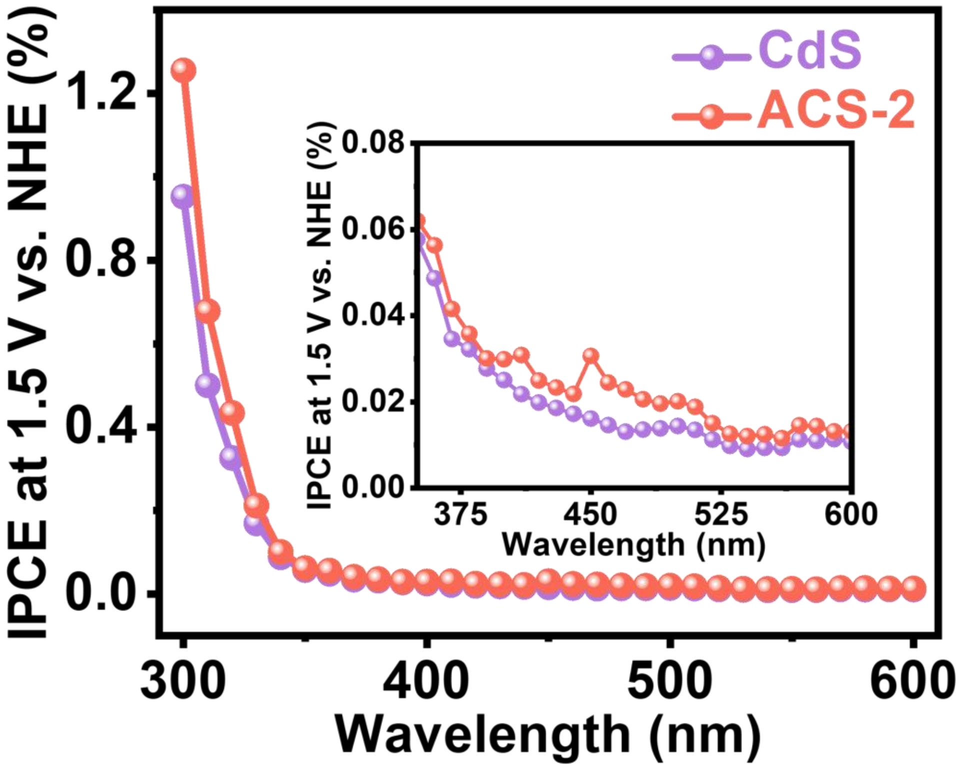

Figure S13. Incident photon-to-electron conversion efficiency (IPCE) of CdS and ACS-2.

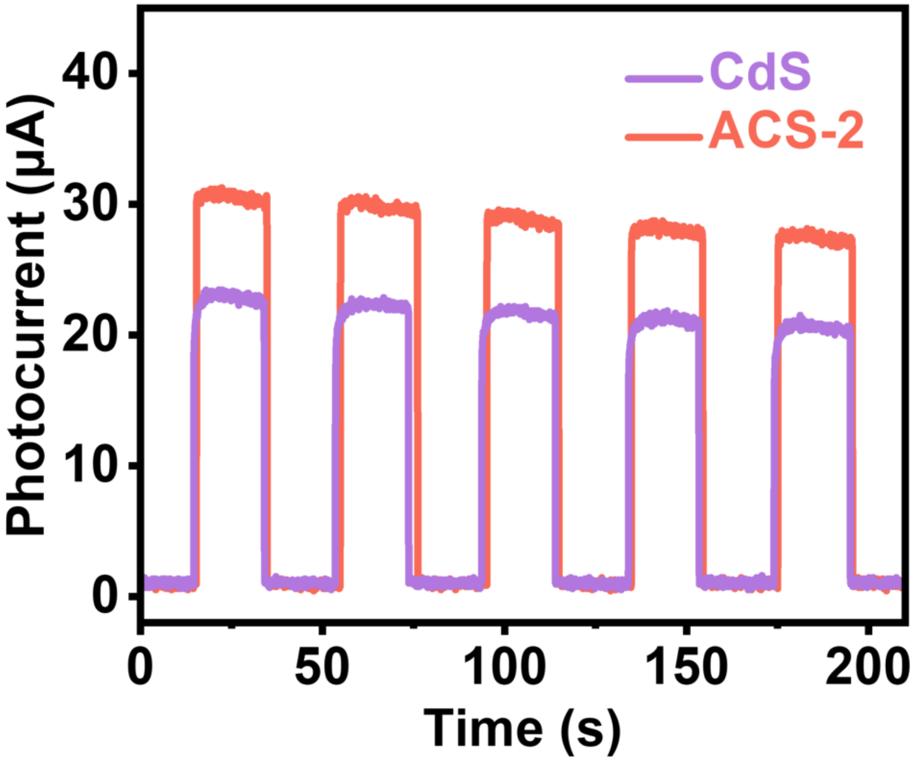

Figure S14. Transient photocurrents of CdS and ACS-2.

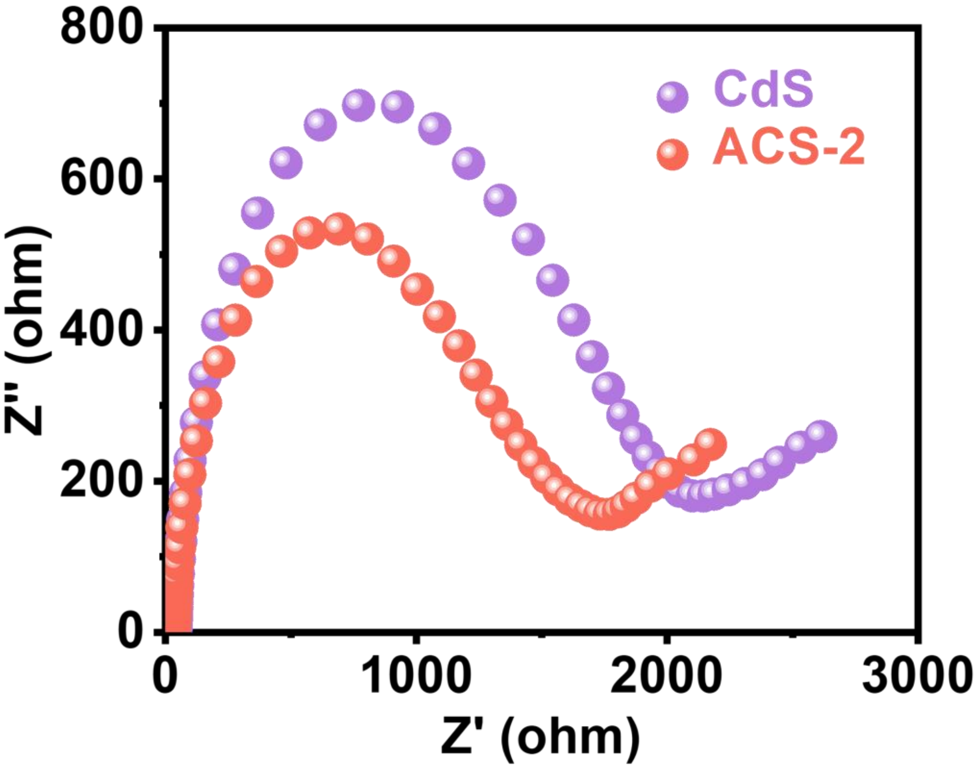

Figure S15. EIS spectra of CdS and ACS-2.

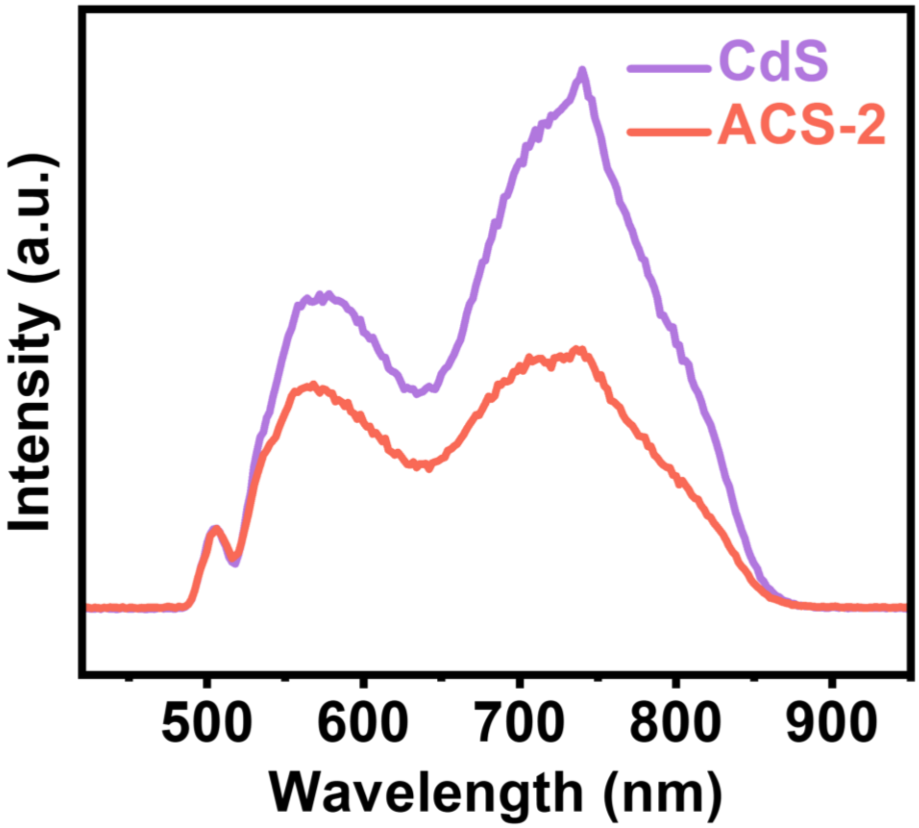

Figure S16. Steady-state PL spectra of CdS and ACS-2.

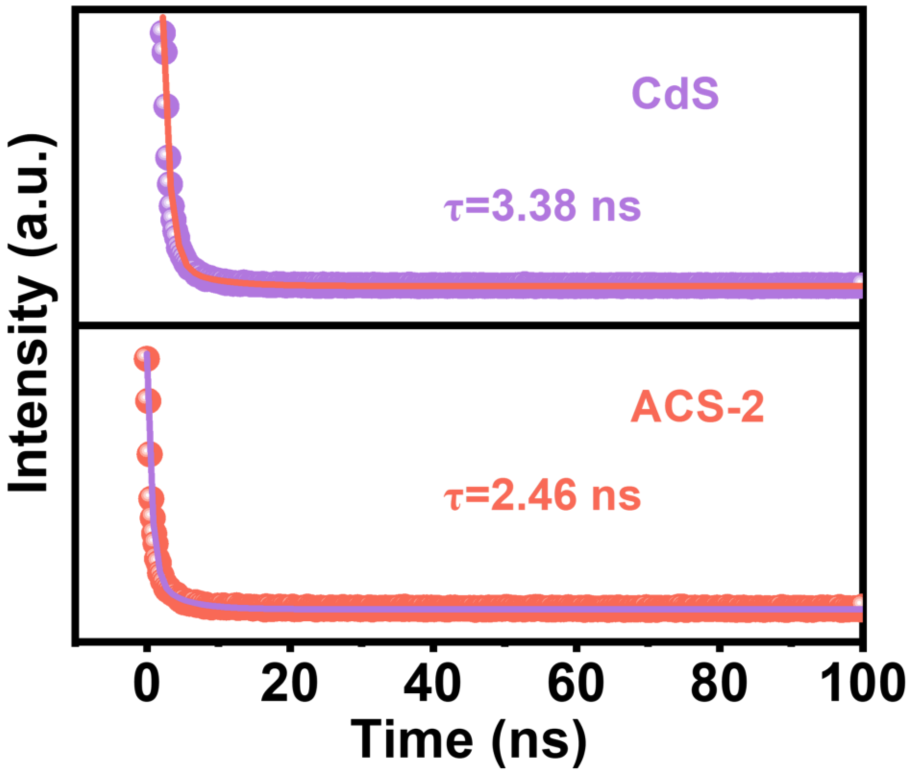

Figure S17. Time-resolved PL (TRPL) spectra of CdS and ACS-2.

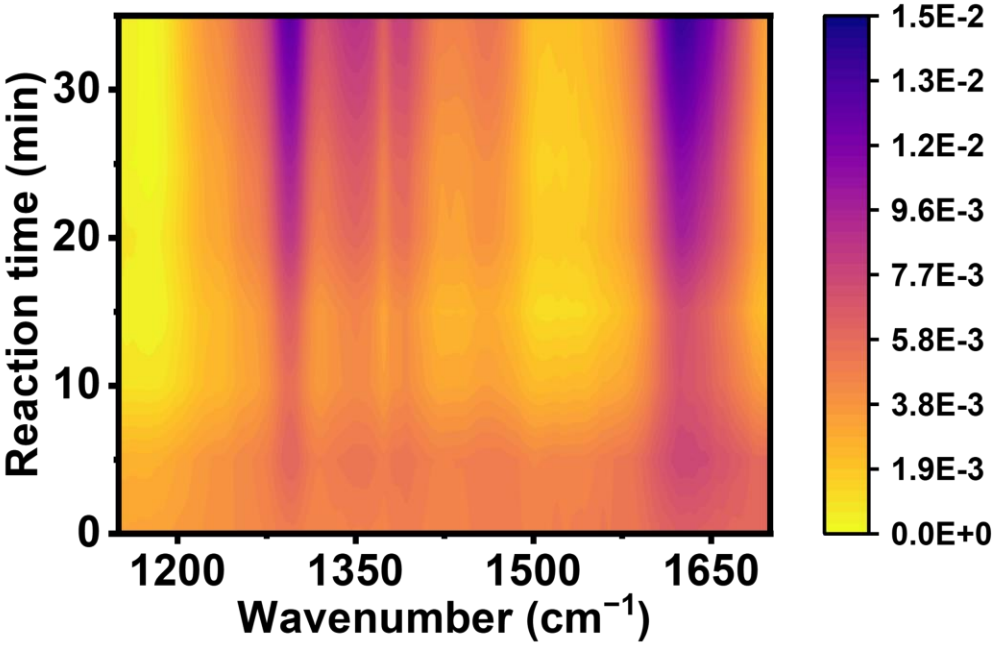

Figure S18. Changes of *in situ* FTIR spectra of ACS-2 with time.

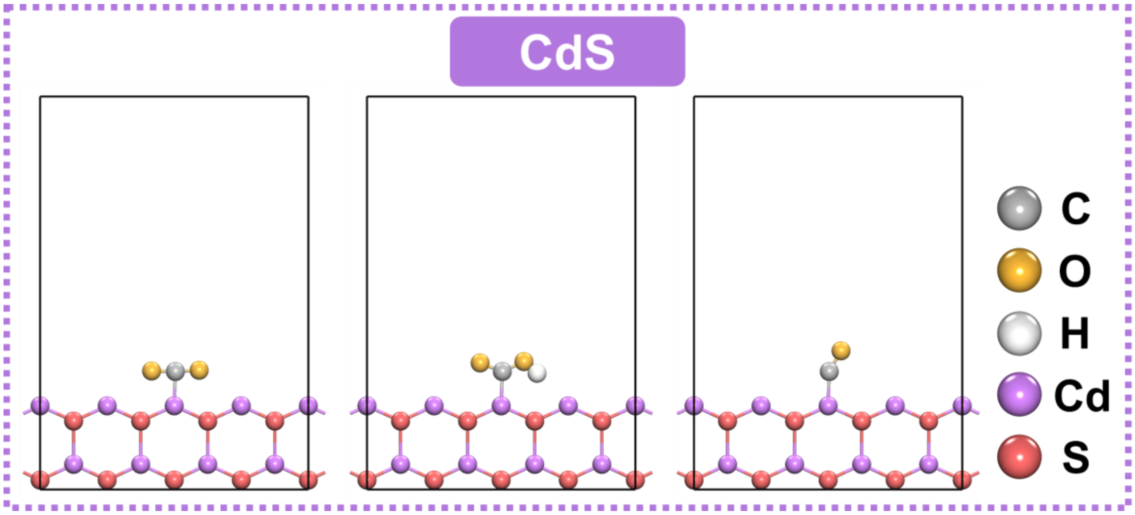

Figure S19. Structural models for calculating the Gibbs free energy of CO_2 activation by CdS.

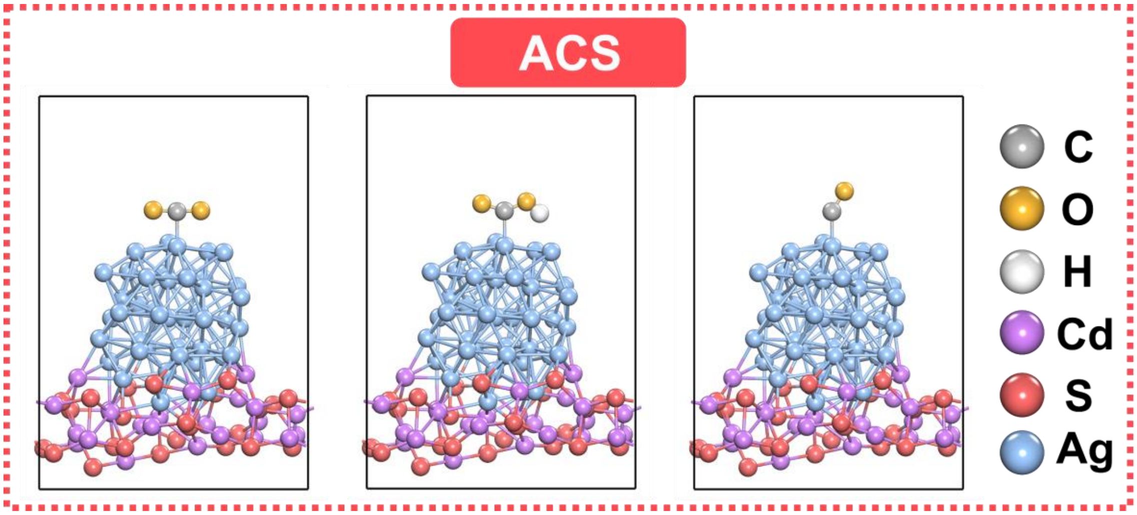

Figure S20. Structural models for calculating the Gibbs free energy of CO_2 activation by ACS.

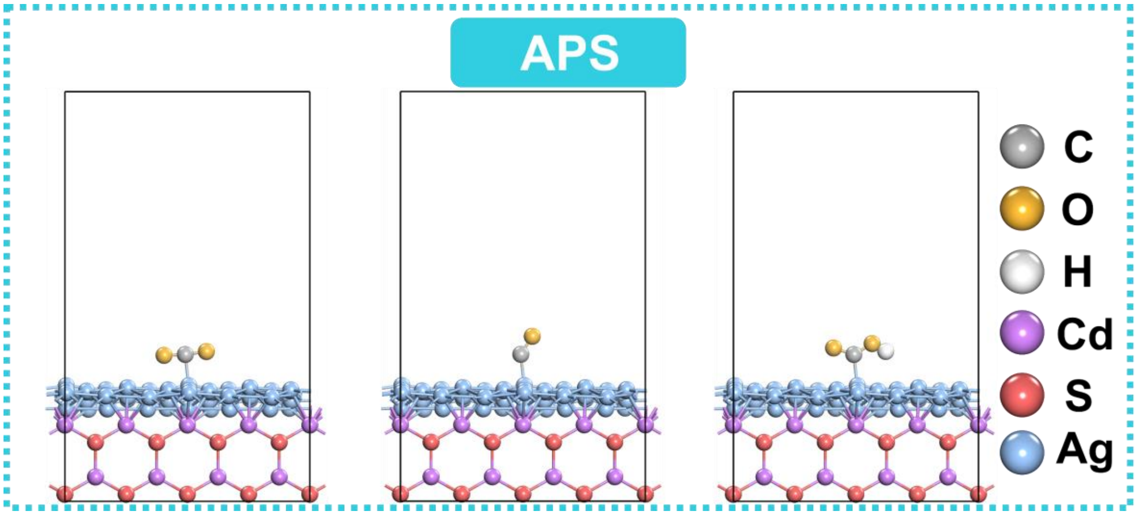

Figure S21. Structural models for calculating the Gibbs free energy of CO_2 activation by APS.

Table S1. Energy changes before and after structural optimization in theoretical computational models.

Models	Before optimization	After optimization
CdS	−111.917086 eV	−117.566066 eV
ACS	−202.913885 eV	−252.232702 eV
APS	−227.318922 eV	−247.512990 eV

Table S2. Comparison of the reaction conditions and characteristics with other photocatalysts for photocatalytic CO_2 conversion.

**Photocatalytic**	**Reaction medium**	**Main products (μmol g^{-1} h^{-1})**	**Reference**
Ag_{44} NCs/CdS	TEOA, MeCN, H_2O, 10 mg catalyst	624.65 (CO)	This work
Cu:CdS QDs	TEOA, H_2O, 20 mg catalyst	30.3 (CO)	Inorg. Chem. 2024, 63, 2234−2240
Mg:CdS QDs	TEOA, H_2O, 2 mg catalyst	45.8 (CH_4)	Adv. Funct. Mater. 2025, e09666
Cu/CdS	TEOA, MeCN, H_2O, 2 mg catalys	14.4 (CO)	ACS Catal. 2024, 14, 1468−1479
CuCo-CdS/C	TEOA, MeCN, H_2O, 10 mg catalyst	63.34 (CO), 35.20 (CH_4)	Sep. Purif. Technol. 2025, 376, 133850
CdS@CTF-HUST-1	TEOA, H_2O, 10 mg catalyst	168.77 (CO)	Adv. Funct. Mater. 2023, 33, 2308553
CdS/$Ti_3C_2T_x$/Au_x NCs	TEOA, MeCN, H_2O, 10 mg catalyst	117.34 (CH_4)	Chem. Sci. 2024, 15, 13495–13505
Au_{25} NCs/RCN	TEOA, MeCN, H_2O, 10 mg catalyst	111.95 (CO)	Ultrason. Sonochem. 2023, 101, 106653

Table S3. Ag concentrations determined by ICP-OES.

Samples	Concentration	Sample mass	Weight percent
**ACS-1**	2.6 mg/L	50.0 mg	0.52wt%
**ACS-2**	15.7 mg/L	46.7 mg	3.36wt%
**ACS-3**	30.7 mg/L	50.0 mg	6.14wt%
**APS**	16.6 mg/L	50.0 mg	3.32wt%

Table S4. TRPL lifetimes of CdS and ACS-2.

Samples	τ_1 (ns)	A_1 (%)	τ_2 (ns)	A_2 (%)	τ_{ave} (ns)
**CdS**	3.78	18.2	0.67	81.8	3.38
**ACS-2**	2.71	17.0	0.43	83.0	2.46

Table S5. fs-TAS lifetimes of CdS and ACS-2 under GSB of 503 nm.

Sample	τ_1 (ps)	A_1 (%)	τ_2 (ps)	A_2 (%)	τ_3 (ps)	A_3 (%)	τ_{ave} (ps)
**CdS**	19.5	0.7	160.4	18.6	1218.8	80.7	1187.4
**ACS-2**	48.7	3.3	438.5	96.0	0.5	0.7	437.0

Table S6. fs-TAS lifetimes of CdS and ACS-2 under GSB of 514 nm.

Sample	τ_1 (ps)	A_1 (%)	τ_2 (ps)	A_2 (%)	τ_3 (ps)	A_3 (%)	τ_{ave} (ps)
**CdS**	28.6	0.8	176.2	24.3	1209.0	74.9	1162.0
**ACS-2**	506.7	48.3	506.0	48.4	52.6	3.3	504.7

Table S7. Energies (eV) of the CdS surface and corresponding groups.

CdS	Slab (*)	*CO_2	*COOH	*CO	CO
E/eV	−117.57	−140.79	−143.42	−133.19	-
ΔE/eV	0.00	−0.24	0.75	−0.60	0.83
G/eV	0.00	0.33	1.16	0.72	0.64
ΔG/eV	0.00	0.33	0.84	−0.45	−0.08

Table S8. Energies (eV) of the ACS surface and corresponding groups.

ACS	Slab (*)	*CO_2	*COOH	*CO	CO
E/eV	−252.23	−275.68	−278.68	−268.08	-
ΔE/eV	0.00	−0.47	0.38	−0.23	1.06
G/eV	0.00	0.10	0.56	0.49	0.64
ΔG/eV	0.00	0.10	0.46	−0.07	0.15

Table S9. Energies (eV) of the APS surface and corresponding groups.

APS	Slab (*)	*CO_2	*COOH	*CO	CO
E/eV	−247.51	−270.92	−273.81	−263.30	-
ΔE/eV	0.00	0.14	0.58	−0.32	1.00
G/eV	0.00	0.14	0.72	0.55	0.64
ΔG/eV	0.00	0.14	0.58	−0.17	0.09
